# Ultrasound elastography in the evaluation of thyroid nodules: evolution of a promising diagnostic tool for predicting the risk of malignancy

**DOI:** 10.1590/0100-3984.2018.0084

**Published:** 2019

**Authors:** Pedro Henrique de Marqui Moraes, Rosa Sigrist, Marcelo Straus Takahashi, Marcelo Schelini, Maria Cristina Chammas

**Affiliations:** 1 Instituto de Radiologia do Hospital das Clínicas da Faculdade de Medicina da Universidade de São Paulo (InRad/HC-FMUSP), São Paulo, SP, Brazil.; 2 Instituto da Criança do Hospital das Clínicas da Faculdade de Medicina da Universidade de São Paulo (ICr/HC-FMUSP), São Paulo, SP, Brazil.

**Keywords:** Thyroid gland, Elasticity imaging techniques/methods, Ultrasonography/methods, Thyroid nodule/diagnostic imaging, Shear wave elastography, Glândula tireoide, Técnicas de imagem por elasticidade/métodos, Ultrassonografia/métodos, Nódulo da glândula tireoide/diagnóstico por imagem, Elastografia *shear wave*

## Abstract

The elastic properties of tissue have always been of interest in clinical practice. In the past, the identification of structures that were stiffer on physical palpation would raise the suspicion that “there was something wrong”. With the development and advancement of medicine, there proved to be a true correlation in the prediction of malignancy of a lesion: malignant disease tends to stiffen the affected tissue, either by increased cell proliferation or fibrosis. Palpation is the oldest method for the detection of thyroid nodules, which is informed by the knowledge that malignant thyroid lesions tend to be much harder than benign ones. Unfortunately, palpation is a highly subjective method that is dependent on the size and location of the lesion, as well as on the skill of the physician. In cases where these nodules are very small or are located in deep regions, their detection by palpation is difficult or even impossible. In addition, although a malignant lesion differs in terms of elasticity, it may not have echogenic properties, preventing its detection by conventional ultrasound. Imaging that indicates the stiffness or deformation of tissues, through the use of ultrasound elastography techniques, adds new information related to their structural formation. In this article, we review the basic physical principles of elastography and the evolution of the method for the evaluation of thyroid nodules, as well as the limitations of and future perspectives for its use.

## Abstract (pt)

A propriedade elástica dos tecidos sempre foi motivo de interesse na prática clínica. A palpação física de estruturas mais rígidas levantava a suspeita de que “havia algo estranho” e isso se mostrou, com o desenvolvimento e avanços da medicina, uma correlação verdadeira na predição de malignidade de uma lesão: a doença maligna tende a enrijecer o tecido acometido, quer por proliferação celular aumentada ou por fibrose. A palpação é o método mais antigo para a detecção de nódulos tireoidianos. Lesões tireoidianas malignas tendem a ser muito mais duras do que as benignas. Infelizmente, a palpação é um método altamente subjetivo e depende do tamanho e localização da lesão e da habilidade do médico. Nos casos em que esses nódulos são muito pequenos ou estão localizados em regiões profundas, a sua detecção por palpação é dificultada ou até mesmo impossível. Além disso, mesmo sendo elasticamente diferente, a lesão pode não apresentar propriedades ecogênicas, impedindo a sua detecção por ultrassonografia convencional. A imagem da rigidez ou deformação desses tecidos acrescenta novas informações relacionadas à sua formação estrutural por meio das técnicas de elastografia. Revisamos, neste artigo, os princípios físicos básicos e a evolução da elastografia para avaliação de nódulos tireoidianos, bem como as limitações e perspectivas futuras do uso dessa técnica.

## INTRODUCTION

Ultrasound elastography techniques measure the elasticity of tissues in order to produce qualitative and quantitative information that can be used for diagnostic purposes in various diseases. The measurements are acquired in specialized imaging modes that can detect tissue stiffness in response to an applied mechanical force (compression or shear wave). In general, ultrasound elastography techniques can be divided into compression imaging methods, which use internal or external deformation stimuli, and shear wave imaging methods, which use ultrasound-generated shear wave stimuli^(^^1^^)^.

Elastography was invented in 1990, having since been undergoing modifications and technological advances that make it increasingly efficient and reproducible. Ultrasound elastography of the liver, for the noninvasive assessment of liver fibrosis, is already a reality in clinical diagnostic practice. Concomitantly, there have been recent advances in the application of elastography for imaging of the breast, prostate, lymph nodes, and thyroid^(^^2^^)^.

## PRINCIPLES AND TECHNIQUES OF ULTRASOUND ELASTOGRAPHY

**Figure 1:**
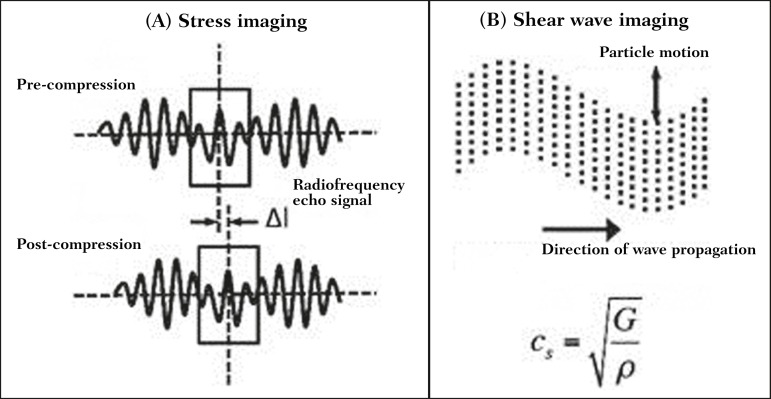
Physics of ultrasound elastography and the methods of measurement. In stress imaging (**A**), the tissue displacement is measured by correlating the radiofrequency echo signals between search windows (boxes) in the pre- and post-compression states. In the shear wave image (**B**), the particle motion is perpendicular to the direction of wave propagation, with shear wave velocity (*c*_s_) related to the shear modulus (G).

There are two elastic moduli that are useful for elastography, categorized by the deformation method employed: Young’s modulus of elasticity, which is defined as the level of external stress required to produce a normal degree of stress perpendicular to the surface; and the shear modulus, which is defined as the shear (dynamic) stress required to generate shear waves tangential to the surface. The shear waves have particle motion perpendicular to the direction of wave propagation, which creates greater differences between the tissues, providing adequate tissue contrast for the elastography measurements (Figure 1).

In the early techniques of elastography, known as strain or compression elastography, it was necessary for the operator to press the ultrasonic probe against a certain region of the body, evaluating the degree of deformation of the various internal structures of the tissues, their deformation being inversely proportional to their stiffness. Young’s modulus was then calculated in order to categorize the image by the ratio between the tissue deformation generated in the different acoustic modes and the force applied on the tissues. That method provides qualitative measures of nodule stiffness^(^^3^^)^. Compression elastography is imprecise and has a number of biases^(^^3^^,^^4^^)^: manual compression is operator-dependent and has a long learning curve; the technique is of limited utility in the evaluation of deep nodules and nodules near the carotid artery, the pulsation of which can cause variations in the measurements; nodules with a diameter > 3 cm or located in the isthmus might not be adequately compressed; and nodules with large cystic areas or coarse “eggshell” calcifications can also defy evaluation.

With the advent of the acoustic radiation force impulse (ARFI) technique, it became possible to evaluate the mechanical properties of the tissues through the application of an acoustic (dynamic) radiation force generated by the ultrasound apparatus (in conjunction with the acoustic wave propagation in the different modes), which made it no longer necessary for the operator to apply manual force on the transducer. As the acoustic frequency increases, there is a displacement of the analyzed tissue, and this displacement generates a shear wave in parallel with and perpendicular to the axis of application of the force. By measuring how fast the wave reaches various lateral positions^(^^5^^)^, the degree of stiffness of the tissue studied is inferred quantitatively (Figure 1).

**Figure 2:**
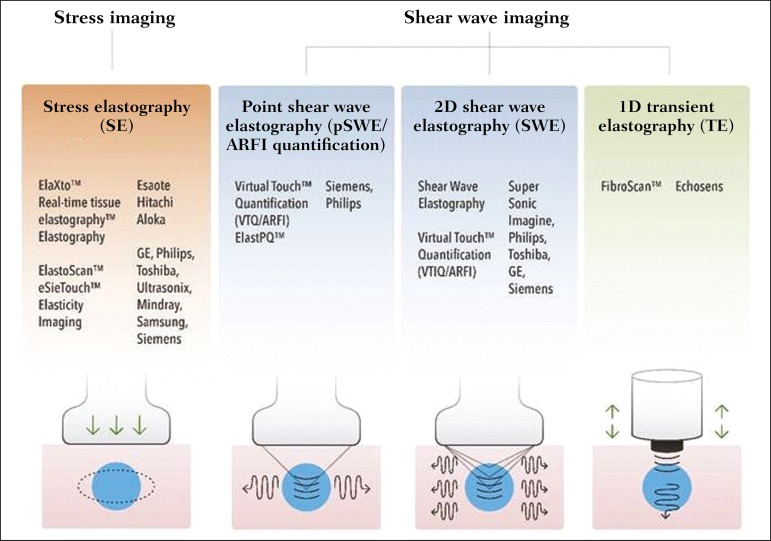
Ultrasound elastography techniques. The elastography techniques currently available can be categorized by the physical quantity measured: compression imaging (**A**) and shear wave imaging (**B**). Excitation methods include quasi-static mechanically induced displacement via active external compression or passively induced physiological movement (in orange), dynamic compression induced by “beating” the transducer on the tissue surface to produce shear waves (in green), and dynamic ultrasound, defined as displacement of the induced tissue and the generation of shear waves through ARFI excitation (in blue).

A number of systems employ the ARFI technique (Figure 2). For example, one-dimensional transient elastography, popularly known by the trade name FibroScan (Echosens, Paris, France), is widely used for the evaluation of liver fibrosis^(^^6^^)^. In addition, point shear wave elastography (pSWE) is a technique in which ARFI is used in order to induce perpendicular tissue displacement at a focal location (single point), producing shear waves by the absorption of acoustic energy^(^^5^^)^. In pSWE, the shear wave velocities perpendicular to the excitation plane are measured and reported directly (in m/s) or converted to Young’s modulus (in kPa), to provide a quantitative estimate of the elasticity of the tissue. Another such technique is two-dimensional shear wave elastography (2D-SWE), which employs ARFI in several focal zones, creating a quasi-cylindrical shear wave, thus allowing real-time 2D monitoring for direct determination of shear wave velocity (in m/s) or calculation of Young’s modulus (in kPa) and the generation of quantitative elastograms. The advantages of 2D-SWE include the real-time visualization of a quantitative color elastogram superimposed on a B-mode image^(^^7^^)^, providing information regarding anatomical features and tissue stiffness that guide the operator. This technique with multiple ARFI focal zones is now referred to as 2D-SWE or just SWE^(^^8^^,^^9^^)^.

## APPLICATION OF ELASTOGRAPHY IN THYROID NODES

With the advent of ultrasound and its progressive improvement, the proportion of thyroid nodules identified in the general population has grown considerably, reaching up to 67% in prospective studies of randomly selected patients^(^^10^^,^^11^^)^. The gold standard for the preoperative assessment of thyroid nodules is fine-needle aspiration biopsy (FNAB) with subsequent cytological analysis. The objective of the FNAB is to distinguish between thyroid nodules with a higher risk of malignancy, for which surgery is indicated, and those that should be followed clinically.

Despite the high prevalence of thyroid nodules, only 4-8% of the nodules sampled by FNAB are malignant^(^^12^^,^^13^^)^. However, it would be unfeasible to perform FNAB of all thyroid nodules identified on ultrasound, in any health care system. Therefore, criteria for malignancy on conventional ultrasound (mode B) and Doppler ultrasound images have been established. The criteria for malignancy risk on Doppler ultrasound imaging include the following: nodule hypoechogenicity (markedly hypoechoic nodules have even greater association with malignancy); the absence of a peripheral hypoechoic halo; the presence of microcalcifications; an anteroposterior diameter greater than the transverse diameter; irregular contours; and poorly defined margins. On Doppler flow studies, nodules with greater vascularity in the center than at the periphery are more likely to be malignant, as are those in which the arteries that nourish the nodule have high resistance indices^(^^14^^)^.

There are cases in which FNAB is not able to classify a thyroid nodule as benign or malignant, such as those of follicular thyroid adenoma of follicular thyroid carcinoma^(^^15^^)^. In 15-30% of cases, the FNAB findings are considered nondiagnostic or inconclusive^(^^12^^)^. Although repeating the FNAB provides conclusive in most cases, inconclusive results are again obtained in up to 50% of nodules with nondiagnostic initial cytology findings and in 38.5-43.0% of those with indeterminate initial cytology findings^(^^12^^)^. Although some inconclusive FNAB results are attributable to technical factors such as insufficient sampling, a subset are attributable to the less easily corrected dilemma of follicular neoplasms, which can account for 6.7% of the total FNAB results or 22.0% of the inconclusive results^(^^13^^)^.Although 15-30% of follicular neoplasms are malignant, requiring total thyroidectomy, malignancy is difficult to determine by FNAB or even by frozen section histological analysis^(^^13^^,^^16^^)^.

In recent decades, thyroid nodules have been evaluated with ultrasound elastography in order to distinguish between benign and malignant nodules prior to histological analysis, thus potentially reducing the number of patients who require surgical intervention, with all of its comorbidities and associated costs.

The guidelines for ultrasound elastography of the thyroid gland describe certain limitations of the method, such as the fact that calcifications within a nodule, which are common, can hinder the evaluation of nodule stiffness, as can peripheral calcifications, which can impede the passage of the ARFI into the deeper regions of the nodule. In addition, nodules with extensive cystic areas should be carefully examined by compression elastography, because those areas can generate artifacts^(^^8^^)^.

### Elastography of the thyroid gland by the compression method

**Figure 3:**
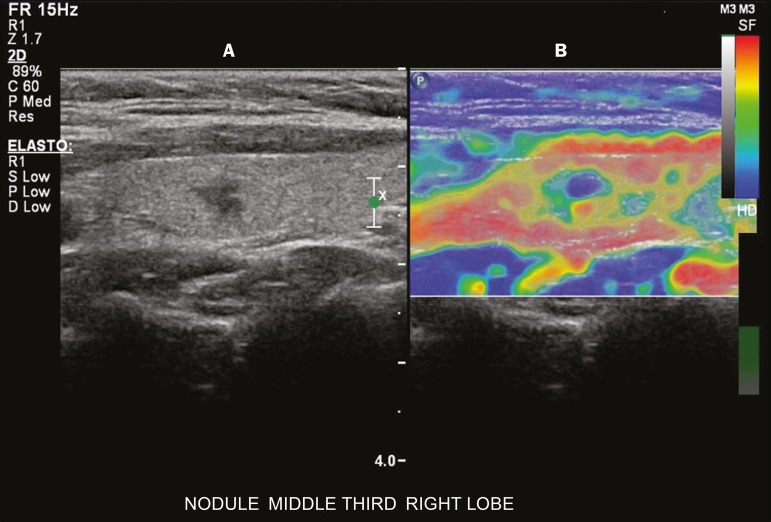
Photograph of B-mode ultrasound (**A**) and color-coded elastogram (**B**) of a thyroid nodule in the right lobe during compression elastography. The nodule appears hypoechoic with poorly defined contours in the anatomical B-mode ultrasound image. The elastogram shows normal thyroid tissue encoded with red color (soft tissue) and the nodule with blue staining (stiff tissue), suggesting a malignant nodule. This was confirmed by histology, which showed papillary thyroid carcinoma.

Compression elastography imaging studies of the thyroid gland can be classified by the types of stimuli and by the classification system employed. The most common stimulus is the external compression applied by the operator through the ultrasound transducer (Figure 3). An alternative technique, using carotid artery pulsation as a physiological stimulus to induce thyroid movement, has produced encouraging results^(^^18^^)^.

There are two established qualitative elasticity scores: the Asteria score^(^^16^^)^, which uses a scale from 1 to 4; and the Rago score^(^^19^^)^, which uses a scale from 1 to 5. The Asteria score divides nodules into four classes, by tissue stiffness, soft nodules being given a score of 1, nodules with an intermediate degree of stiffness being given a score of 2 or 3, and stiff nodules being given a score of 4^(^^16^^)^. In the Rago scoring system^(^^19^^)^, nodules are scored from 1 (same elasticity throughout) to 5 (without elasticity in the nodule or in the area showing posterior shadowing). In both scoring systems, the pattern of elastography is compared with the cytological findings after FNAB or with the histological findings after surgical resection (confirmation or exclusion of malignancy).

Studies using compression elastography to evaluate thyroid nodules have produced contradictory results. A meta-analysis, including a collective total of 639 thyroid nodules, showed that compression elastography was useful for evaluating malignancy, with a mean sensitivity of 92% and a mean specificity of 90%^(^^20^^)^. Those findings were challenged by the results of a recent retrospective study of 703 nodules, which showed that the sensitivity of deformation measurements was 15.7% when the 5-point Rago score was applied and 65.4% when the 4-point Asteria score was applied, both lower than the 91.7% achieved with B-mode ultrasound^(^^7^^)^. More recently, a prospective study of 912 nodules found that compression elastography was superior to B-mode ultrasound for predicting malignancy, with a sensitivity of 80.2% and a specificity of 70.3%^(^^21^^)^.

Given that B-mode ultrasound and compression elastography provide independent measurements, the combination of the two methods would hypothetically be superior in predicting malignancy. That hypothesis was tested by Trimboli et al.^(^^22^^)^, who found that the combination of the two modalities had a 97% sensitivity and a 97% negative predictive value, greater than the use of the elastography alone (81% and 91%, respectively) or B-mode ultrasound alone (85% and 91%, respectively). In contrast, Moon et al.^(^^7^^)^ found that the combination of compression elastography measurements and B-mode ultrasound characteristics was less accurate than was B-mode ultrasound alone for the identification of malignancy (Table 1). Those contradictory results might be attributable to the differences across studies in terms of the techniques used, the calibration of the equipment, divergences from the gold standard (cytology or histology) for comparative statistical analysis, and the exclusion criteria. Specifically, the proportion of malignant thyroid nodules differs among studies, ranging from the 9.4% reported by Azizi et al.^(^^21^^)^ to the 31.0% reported by Moon et al.^(^^7^^)^. Additional prospective studies, involving larger samples, are needed in order to assess the clinical value of compression elastography in the characterization of thyroid nodules.

**Table 1:** Summary of studies evaluating compression elastography for the identification of malignant thyroid lesions.

Study	Patients (n)	Lesions (n)	Malignant lesions(n)	Technique	Parameter	Sensitivity (%)	Specificity (%)
Bojunga et al.^(20)^	530	639	153	Strain elastography	Strain ratio	92.0	90.0
Moon et al.^(7)^	676	703	217	Strain elastography	Strain ratio (4-point scale)*	65.4	58.2
					Strain ratio (5-point scaled)†	15.7	95.3
Azizi et al.^(21)^	706	912	86	Strain elastography	Strain ratio	80.2	70.2
Trimboli et al.^(22)^	446	498	126	Strain elastography	Strain ratio	81.0	62.0

* Asteria score^(16)^;

† Rago score^(19)^.

A number of specific limitations of compression elastography have been highlighted in the literature^(^^23^^,^^24^^)^. For example, manual external compression is subject to interoperator variability. In addition, the nonlinearity of tissue stiffness results in higher stiffness values at higher degrees of compression. Furthermore, fibrosis can increase the stiffness of benign and malignant nodules. Moreover, small sample sizes can imply a selection bias. Finally, there is a lack of standardization of the technique parameters (e.g., color scales and cutoff values).

### Elastography of the thyroid gland by SWE

**Figure 4:**
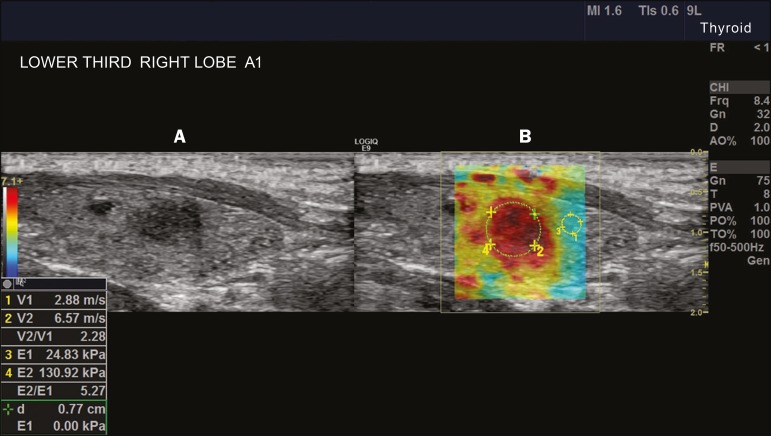
B-mode ultrasound (**A**) showing a hypoechoic thyroid nodule with poorly defined margins in the right lobe, suggesting a malignant etiology. The corresponding color-coded elastogram (**B**) shows greater stiffness in the ROI within the nodule than in the surrounding normal parenchyma (130.92 kPa vs. 24.83 kPa), suggesting that the nodule is malignant. A subsequent biopsy confirmed the diagnostic hypothesis of papillary carcinoma.

Unlike compression elastography, SWE provides quantitative measurements of the elasticity of thyroid nodules (Figure 4).

#### pSWE

A number of recent meta-analyses of pSWE have shown promising results^(^^25^^-^^28^^)^, with similarities among the studies. In the largest meta-analysis of pSWE studies to date, Zhan et al.^(^^25^^)^ evaluated data related to a collective total of 2436 thyroid nodules. The authors found that pSWE was useful in distinguishing between benign and malignant nodules, with a mean sensitivity of 80% and a mean specificity of 85%. In another meta-analysis of pSWE studies, including a collective total of 1617 thyroid nodules, Dong et al.^(^^28^^)^ found that the method had a pooled sensitivity of 86.3% and a pooled specificity of 89.5%.

#### 2D-SWE

Recent studies employing the 2D-SWE method have shown promise for making the distinction between benign and malignant thyroid nodules, with less bias and greater reproducibility. Hang et al.^(^^29^^)^, Sebag et al.^(^^30^^)^, Veyrieres et al.^(^^31^^)^, and Kim et al.^(^^32^^)^ found very similar results for the nodule elasticity index cutoff values above which the risk of carcinoma is increased, those cutoffs ranging from 62 kPa to 69 kPa. In comparison with the post-FNAB cytology and the histology for cases of malignancy or suspected malignancy, those cutoff values showed a sensitivity ranging from 66.6% to 85.2% and a specificity ranging from 71.1% to 93.9% (Table 2).

**Table 2:** Summary of studies of 2D-SWE evaluating nodule elasticity index cutoff points above which the risk of carcinoma is increased.

Study	Patients (n)	Lesions (n)	Malignant lesions (n)	Technique	Equipment	Parameter	Cutoff (kPa)	AUC	Sensitivity (%)	Specificity (%)	PPV (%)	NPV (%)
Hang et al.^(29)^	244	289	170	2D-SWE	Aixplorer*	YM (kPa) mean	69	0.76	75.3	75.3	—	—
Sebag et al.^(30)^	93	146	29	2D-SWE	Aixplorer*	YM (kPa) mean	65	0.93	85.2	85.2	80.0	95.9
Veyrieres et al.^(31)^	148	297	35	2D-SWE	Aixplorer*	YM (kPa) mean	66	0.85	80.0	80.0	—	97.1
Kim et al.^(32)^	99	99	21	2D-SWE	Aixplorer*	YM (kPa) mean	62	0.76	66.6	66.6	40.6	85.7

AUC, *area under the curve*; PPV, positive predictive value; NPV, negative predictive value; YM, Young’s modulus.

* SuperSonic Imagine; Aix-en-Provence, France.

A recent prospective study examined whether 2D-SWE could distinguish between benign and malignant follicular neoplasms^(^^12^^)^. In that study, 35 patients with thyroid nodules in whom follicular neoplasms had been diagnosed by FNAB were evaluated preoperatively with B-mode ultrasound and 2D-SWE. The authors found that, although B-mode ultrasound characteristics were not predictive of follicular malignancy, higher estimated Young’s modulus values were associated with malignancy in follicular nodules (area under the curve = 0.81, cutoff = 22.3 kPa, sensitivity = 82%; specificity = 88%, positive predictive value = 75%, negative predictive value = 91%).

In comparison with compression elastography, the SWE technique is subject to less interoperator variability and is more reproducible. However, the lack of standardization of the technique and the different calibrations offered by the various manufacturers continue to represent an obstacle to the dissemination of the modality.

In one recent study^(^^33^^)^, the findings obtained with all three of the main elastography techniques-compression elastography, pSWE, and 2D-SWE-were compared with those of the cytological and histological analyses. The authors concluded that the techniques that employ the ARFI technique presented superior results in the prediction of malignancy risk, with a sensitivity, specificity and negative predictive value of 90%, 79%, and 98%, respectively. The pSWE and 2D-SWE techniques showed significantly better area under the curve values than did compression elastography (*p* = 0.008). The authors attributed that superiority to the greater operator dependence of compression elastography techniques. In the literature, many different cutoff values have been proposed for the distinction between benign and malignant thyroid nodules with the three methods of elastography. These discrepancies are explained by the fact that there are various criteria for selecting the most appropriate cutoff values for a diagnostic test. The calculation of a cutoff value depends on the prevalence of malignant thyroid nodules, the study population, and the nodule sample size. No consensus has been established to date.

### Reproducibility

The first studies assessing interobserver agreement for compression elastography in the evaluation of thyroid cancer demonstrated that the results obtained with compression elastography are inferior to those obtained with conventional ultrasound^(^^34^^)^. Since then, various other studies have reported the reproducibility of compression elastography and SWE. Most of those studies showed substantial or near-perfect interobserver agreement. The remarkable improvement in interobserver agreement can be explained by the improvements in compression elastography devices, the advent of the real-time compression monitoring reducing over- or under-compression, which previously skewed the elastography scoring. In addition, the use of SWE has eliminated the bias of manual compression, resulting in greater reproducibility^(^^34^^)^.

## CONCLUSION

Elastography has proven useful as an ancillary tool for risk stratification in thyroid nodules. The evolution of the technique has improved its reproducibility, and recent studies have shown that 2D-SWE is a promising technique for the identification of malignant nodules, either before FNAB or after an indeterminate cytological result. In Brazil, compression elastography is the technique most widely used for superficial structures, including the thyroid gland. At some centers, SWE is performed as a complementary method in the analysis of the malignancy risk of thyroid nodules.

Future prospects for the use of elastography in clinical practice call for standardization of the technique. Thus, it will be possible to compare values across studies and to develop new solutions for current technical limitations.
